# A North–South-South partnership in higher education to develop health research capacity in the Democratic Republic of the Congo: the challenge of finding a common language

**DOI:** 10.1186/s12961-021-00728-8

**Published:** 2021-05-07

**Authors:** Christiane Horwood, Sphindile Mapumulo, Lyn Haskins, Vaughn John, Silondile Luthuli, Thorkild Tylleskär, Paulin Mutombo, Ingunn M. S. Engebretsen, Mala Ali Mapatano, Anne Hatløy

**Affiliations:** 1 Centre for Rural Health, School of Nursing and Public Health, University of KwaZulu-Natal, Durban, South Africa; 2 School of Education, University of KwaZulu-Natal, Pietermaritzburg, South Africa; 3 Centre for International Health, University of Bergen, Bergen, Norway; 4 Kinshasa School of Public Health, University of Kinshasa, Kinshasa, Democratic Republic of Congo; 5 Fafo Institute for Labour and Social Research, Oslo, Norway

**Keywords:** English as a medium of instruction, Higher education, Low- and middle-income countries, Research capacity, Partnerships, North–South-South, Nutrition, Epidemiology, Democratic Republic of the Congo, Africa

## Abstract

**Background:**

Globally, increasing numbers of higher education institutions (HEIs) in non-English-speaking countries have adopted English as a medium of instruction (EMI), because of the perception that this provides opportunities to attract high-calibre students and academic staff, and engage with the international research community. We report an evaluation of a North–South-South collaboration to develop health research capacity in the Democratic Republic of the Congo (DRC) by establishing a postgraduate programme in nutritional epidemiology at the Kinshasa School of Public Health (KSPH), where EMI was adopted. We report experiences and perceptions of stakeholders, facilitators and students about using EMI.

**Methods:**

In-depth qualitative interviews were conducted between October and December 2019 among convenience sampled stakeholders (8), facilitators (11) and students (12) involved in the programme from all three partner institutions (University of Kinshasa; University of KwaZulu-Natal, South Africa; University of Bergen, Norway). Interviews were conducted in participants’ language of preference (English or French), audio-recorded, transcribed verbatim and translated into English when required. Analysis employed a thematic approach.

**Results:**

Most participants viewed EMI positively, reporting that studying in English created opportunities to access relevant literature, improve interactions with the scientific community and advance their careers. As a result of adopting EMI, some students had opportunities to present research findings at international conferences and publish their research in English. English-speaking researchers from partner institutions were able to participate in supervision of students’ research. However, inadequate English competency, particularly among students, was challenging, with some students reporting being unable to understand or interact in class, which negatively affected their academic performance. Further, EMI created barriers at KSPH among academic staff who were not proficient in English, leading to poor participation among non-English-speaking staff and lack of integration with other postgraduate programmes. Participants suggested additional English language support for EMI.

**Conclusion:**

Partnerships between HEIs could be a powerful tool to develop research capacity in low-income countries in line with United Nations Sustainable Development Goals. EMI could be a solution to language barriers faced by many such partnerships, but wide-ranging support to develop English proficiency among staff and students is essential to ensure that the challenges do not outweigh the benefits.

## Background

To effectively address the health challenges in low-income countries, it is essential to develop context-specific research capacity in settings where skills are profoundly lacking [1]. Low-income countries require local research skills to explore factors underlying poor health and nutrition and develop solutions to address local challenges. Research capacity-building has the potential to inform and develop strong health service delivery systems using effective evidence-based health interventions. The United Nations Sustainable Development Goals (SDGs) support the use of global partnerships to promote sustainable development (SDG17) [2], and such partnerships are well placed to address the challenge of developing local research capacity in low-income countries. Aligned with the SDGs, partnerships have been established between higher education institutions (HEIs) in Northern Hemisphere high-income countries (HIC) and Southern Hemisphere low-income counterparts to build or strengthen research capacity in the low-income country [3–5]. Some partnerships, known as North–South partnerships, have been successful in training researchers and improving academic leadership in low-income countries [3, 4, 6–8], but others have been criticized with concerns being expressed that Northern partners being in control of funding and the research agenda can lead to most benefits accruing to the North [6, 7, 9]. Another challenge is that language barriers frequently exist between partners, and the choice of language of instruction has often been a challenge to effective implementation of North–South partnerships [10–13].

The use of English as a medium of instruction (EMI) by HEIs in countries where English is not the home language (or first language) or the national (administrative) language is a widespread phenomenon that continues to increase globally [14–16]. The trend towards English language is not just in higher education but also in commerce, where the perceived economic benefits are so strong that governments in some African countries, notably Rwanda and Burundi, have adopted English as the national language [17, 18]. EMI has been adopted by universities in both high- and low-income countries. In some countries, this is because the home language is not widely spoken outside of the country, but in many multilingual African countries where there is no common language, like the Democratic Republic of the Congo (DRC), an administrative language is adopted as a medium of instruction throughout the country. This is usually a previous colonial language. [19]. HEIs in many countries, including in Europe, Africa and Asia, choose to use EMI for some or all of their study programmes, particularly for postgraduate studies, because of the perception that using EMI presents opportunities for the institution, students and the country as a whole [20]. Using English may allow institutions to access a larger pool of students and academic staff, thereby attracting personnel of high calibre, to attract funds from international organizations and to participate in national and international collaborative academic and research programmes [21]. From the students’ perspective, English language skills may increase employability, allow employment mobility and allow students to access crucial learning resources only available in English. Use of English also enables students to obtain wider access to scientific and research communities and may provide students from disadvantaged backgrounds a pathway to accessing opportunities for international scholarship and research [15]. For these reasons, students, parents, academics and stakeholders in many countries support the use of EMI in HEIs [14, 20, 21]. Although teaching exclusively in English may come across as politically insensitive in countries where English was previously a colonial language, this tension must be balanced by a rights approach and social justice perspective to develop capabilities of students in not only indigenous language but also in global language [22].

However, there is a counterargument about the challenges of using EMI in countries where English is not the home language, with scholars in teaching and learning arguing that the language of instruction should be the home language to effectively equip students with skills and knowledge, and to develop the local language culturally, scientifically and technologically [16, 23]. Further, the learning in global health should proactively address inequity related to the predominance of English language in the discourse and promote the inclusion of non-English-speaking academics [22]. The main concern is that inadequate English skills of both teachers and students could adversely affect the quality of teaching and learning [24]. Teachers must have good command of the language of instruction as well as knowledge of the subject content to effectively interact with students during the teaching process [21]. It is important that lecturers have adequate English proficiency, not only to effectively convey knowledge to students, but also to answer questions and undertake interactive learning activities. An example is the University of Korea, where to ensure English proficiency when promoting EMI, policies were implemented to recruit only staff who were competent in English, and students were required to pass a number of English courses to graduate [14].

Further, EMI can have a negative impact on students’ learning experience and achievements. Although students are often motivated to learn in English to access the perceived benefits, inadequate English proficiency may become a barrier, with the result that these students frequently achieve very little because they are unable to learn effectively [14]. First-year students in Bangladeshi HEIs expressed that learning in English was challenging for them with lack of English competency, particularly among disadvantaged students, affecting them academically and socially [25]. Therefore, EMI has resulted in poor academic performance [26], particularly among students from poor and disadvantaged backgrounds [23]. Learning exclusively in English may therefore fail to provide students from disadvantaged communities with the opportunity to be part of the global economy [27]. When students cannot understand what is being taught, they may opt for memorizing and reciting information rather than gaining an in-depth understanding of the content, which limits their ability to answer questions or develop arguments that require analytical and critical thinking [21, 23].

The use of a bilingual system in institutions where English is not the home language has been supported by some policy-makers, scholars and linguists claiming that a bilingual system is an equitable approach that would benefit students and enhance teaching and learning, thus improving academic performance [16, 23, 27, 28]. However, bilingual learning has disadvantages, particularly because it hinders the ability of students to effectively learn English. In order for students to be proficient in English, they must be taught regularly throughout their educational career and have opportunities to practice and immerse themselves in the language [29].

As a result of these concerns, it has been suggested that HEIs should rather use the home language as the medium of instruction [16, 23, 30]. However, use of home languages at HEIs, particularly in low-income countries where the language is not widely spoken, may compromise the ability of universities to produce high-calibre students to effectively contribute to the socioeconomic development of poor communities [21, 31]. Therefore, the choice of the medium of instruction is likely to be controversial and difficult to resolve at any given institution, especially within the limited time spans of funded, collaborative partnerships between English-speaking countries and countries with another home language. Nevertheless, with longer time horizons, such tensions may be eased as stakeholders learn from the past experiences and implement ways of supporting EMI programmes.

DRC is a multilingual country with over 200 local languages and four regional *lingua franca* (Lingala, Kikongo, Tshiluba and Ki-Swahili) where French is used as the official administrative language in higher education and government but is not the home language for most citizens. English is compulsory for all secondary school students in DRC, providing a basis for using English as a medium of instruction in higher education. DRC has some of the world’s worst nutrition indicators with high levels of both acute and chronic malnutrition, preventing those affected from achieving their potential and leading to a vicious cycle of intergenerational poverty [32]. Thus, improving nutrition is an important priority for socioeconomic development in DRC. In response to this need, a North–South-South collaboration between three universities was established to support the implementation of a new master’s and doctoral programme in nutritional epidemiology at the Kinshasa School of Public Health (KSPH), University of Kinshasa (UNIKIN). English was adopted as the medium of instruction in this programme.

In this paper, we present an evaluation of the nutritional epidemiology programme with a focus on the experiences of stakeholders, facilitators and students regarding the use of EMI, and suggest how the challenges of this approach can be navigated to provide a model for health research capacity development in low- and middle-income countries (LMIC).

### Growing Partnership for Higher Education and Research in Nutritional Epidemiology (GROWNUT)

The GROWNUT project was a collaboration between three HEIs: KSPH at UNIKIN, DRC; Centre for Rural Health (CRH) at the University of KwaZulu-Natal (UKZN), South Africa; and Centre for International Health (CIH) at the University of Bergen (UiB), Norway. The programme was funded by the Norwegian Agency for Development Cooperation (Norad) through the Norwegian Programme for Capacity Development in Higher Education and Research for Development (NORHED). The aim of the collaboration was to build institutional capacity at the KSPH to deliver high-quality postgraduate nutrition education, by developing and implementing a master’s and PhD programme in nutritional epidemiology to improve local research outputs and inform policy-makers. The programme was developed in close collaboration with the National Nutrition programme at the DRC Ministry of Health (PRONANUT) [8].

The GROWNUT project aimed to support key processes and infrastructure to establish the nutritional epidemiology programme, including the development of a rural research site, provision of bursaries for selected students, and development of a new curriculum which employed English as the medium of instruction. This latter requirement was suggested by the partners in DRC because English was seen as the primary language of scientific enquiry. In addition, use of English allowed researchers at partner institutions to contribute to training, mentoring and supervision of students. At the beginning of the programme, students attended a 2-week course on scientific English and 1-week course in English for nutritionists to prepare them for classroom discourse.

The aim was for the master’s and PhD programme to be conducted entirely in English with facilitators from all partner institutions participating in the teaching programme to support local DRC facilitators, who would take over teaching in the final years of the project and after project completion. However, as a result of political unrest in DRC, international partner facilitators were unable to travel after the first 2 years, after which local facilitators undertook all teaching, and the medium of instruction varied between English and French, depending on the preference of the DRC facilitator. Most master’s and PhD students had two supervisors, a main supervisor from DRC and a supervisor from a partner institution.

Between 2014 and 2018, four cohorts of students (total of 41 students) were enrolled in the nutritional epidemiology master’s programme, of whom 40 students have graduated. Six students were enrolled in the PhD programme, two have graduated, two aim to complete in 2021 and two dropped out of the programme. All PhD students were given the opportunity to gain experience and skills, including English skills, at partner universities. Furthermore, 11 students presented their research findings at international conferences, and 11 research articles have been written by GROWNUT PhD students with coauthors from partnering institutions. Theses were written in English, with the exception of six master's students whose inadequate English skills required them to write their theses in French. All degrees were conferred by UNIKIN.

## Methods

### Study design

A qualitative cross-sectional study was used to conduct an internal evaluation of the GROWNUT project during the sixth year of the project. In-depth interviews were conducted among all identified stakeholders, facilitators and supervisors in three partner institutions, and among selected master's and PhD students.

### Recruitment of participants

Participants included (1) stakeholders at collaborating institutions including the funding body, (2) supervisors and facilitators on the GROWNUT nutritional epidemiology programme, and (3) master’s and PhD students.

Stakeholders were identified by project leaders at each institution based on their participation in planning, oversight or management of the project. Stakeholders included representatives from the funding organization (Norad), senior managers from UNIKIN, KSPH, and UiB, representatives from the Ministry of Health in the DRC and representatives from the rural research site.

All facilitators/supervisors who had participated in teaching or supervision of nutritional epidemiology students were asked to participate. A number of facilitator/supervisors were also stakeholders, having been involved in the management of the project as well as participating in teaching and supervision.

Master’s students in the nutritional epidemiology programme were selected to participate from the total of 40 students, including both graduates and those currently enrolled. Three students were selected from each of the four cohorts using a convenience sampling approach, based on availability and willingness to participate, including at least one female student from each cohort. Three PhD students were asked to participate in the study, two of whom had already graduated, and one who was currently registered and had previously graduated from the master’s programme. The two PhD students who had de-registered were excluded from the study, although one was included among the master’s graduates and was able to contribute to the discussion of EMI.

### Study setting

UNIKIN is one of the largest and oldest universities in DRC established in 1954 as Lovanium University, becoming the National University of Zaire, and finally UNIKIN in 1981. It is currently ranked highest among all universities in the DRC with 12 academic divisions and French as the medium of instruction. The KSPH was established in 1984 with the support of the United States Agency for International Development (USAID) and is situated within the medicine division. KSPH has five departments, namely, Biostatistics and Epidemiology; Health Management and Policy; Nutrition; Community Health; and Environment Health. It currently offers five master’s degree programmes which includes Master in Public Health (MPH), Health Economics, Bioethics, and Field Epidemiology and Laboratory Training Programme (FELTP), and, since 2014, Nutritional Epidemiology.

UiB is one of the largest Universities in Norway, established in 1946 and currently ranked second among Norwegian universities. UiB consists of seven faculties housing 60 different specialized departments, centres and institutes. The CIH was launched in 1988 and is situated within the Faculty of Medicine in the Department of Global Public Health and Primary Care. CIH promotes research, education and leadership development with the aim of improving health in LMIC and responding to global health challenges. CIH staff participate and support teaching and training activities including the supervision of master's and PhD students both nationally and internationally.

UKZN is the sixth-largest university in South Africa having been established in 2004 with a merger of the previous University of Natal and University of Durban-Westville, and is currently ranked fourth among South African universities. Spread across five campuses, UKZN has four colleges and 19 schools. CRH is an externally funded research centre established in 1987, situated in the College of Health Sciences in the School of Nursing and Public Health (SONPH). CRH researchers collaborate with South African, African and international partners to promote the health and well-being of people in underserved areas by engaging in implementation science research to strengthen health systems and services.

### Data collection

In-depth interviews (IDI) were conducted by qualified researchers from UKZN who had not participated in the GROWNUT programme (SL, SM). IDI guides were developed to explore the experiences of participants in using English as a medium of teaching, learning and communication within the nutritional epidemiology programme. Interviews were conducted face-to-face (27), via skype (3) or on the telephone (1) depending on the participant’s location.

Interviews were conducted in the participant’s language of preference, either English or French. For those interviews conducted in French, interpreters were used to translate during interviews. All interviews were audio-recorded.

### Data analysis

Interviews were transcribed verbatim and translated into English where required. Researchers listened to a selection of recordings and read the transcripts to ensure that transcripts were accurate and of good quality. Using an inductive thematic approach, data were analysed by two qualified researchers (SL, SM).

Following a thorough reading of all transcripts by the researchers to familiarize themselves with the data, predetermined themes based on the research questions formed the initial coding framework, and additional themes could be added if they emerged. Meetings were undertaken with the research team to finalize the coding framework. Coding was then undertaken by the two researchers working independently using Nvivo 12.3 software. Meetings were held frequently between the two researchers to monitor progress and discuss whether any new themes had emerged.

### Ethical considerations

All three institutions involved in the GROWNUT project obtained ethics approval to conduct the study: University of KwaZulu-Natal Humanities and Social Sciences Research Ethics Committee (HSSREC) (HSS/0258/019); University of Kinshasa School of Public Health Ethics Committee (ESP/CE/247/2019); and Norwegian Centre for Research Data (NSD) (ref. 466503). All participants provided written informed consent to participate. To maintain confidentiality and anonymity, participants were given unique study numbers, and all identifiable information was removed from transcripts prior to data analysis. Students who travelled to KSPH for interviews were given US$5 to cover costs of transportation.

## Results

IDI were conducted with 31 participants, comprising eight stakeholders, 11 facilitator/supervisors, nine master’s students and three PhD students, one of whom had previously graduated from the master’s programme. Two facilitators, one stakeholder and one student were approached to participate but were unavailable during the study period: one KSPH facilitator was replaced by another KSPH facilitator; one facilitator was no longer working at a participating HEI and could not be reached; one UiB stakeholder could not be reached; and one master's student was unable to attend for interview and was replaced by another student. Participant characteristics are shown in Table 1.

**Table 1:** Demographics characteristics of study participants

Students, *n* = 12	
Age (median)	39 (IQR = 11.5)
Gender	
Male	8
Female	4
Occupation	
Physician/medical doctor	9
Academic assistant (KSPH)	3
Level of academic of study	
PhD/doctoral degree	3
Master’s degree	9
Partner university who co-supervised the degree	
UKZN	5
UIB	6
No co-supervisor from a partner university	1
Attended training at partner universities	
UKZN	9
UIB	3
Supervisors/facilitators/stakeholders (*n* = 19)	
Age (median)	54 (IQR 12)
Gender	
Male	9
Female	10
Role in the project	
Stakeholder (includes managers at KSPH/UNIKIN/UIB and at Norad, and community leader from a rural site)	9
Facilitator/supervisor of the GROWNUT programme	10
Current position	
Professor/academic staff	15
Project manager	2
Representative from the Ministry of Health	1
Community leader of rural research site	1
Institution in which based	
UNIKIN	12
UKZN	3
UiB	4

All interviews with Kinshasa-based participants (25) were conducted in person at UNIKIN, DRC, in October 2019. South Africa-based interviews (3) were also conducted in person during October 2019. Interviews with Norwegian facilitators and stakeholders were conducted face-to-face (1), via skype (3) and by telephone (1) between October 2019 and December 2019.

The themes presented were predetermined based on questions from the IDI guide.

### Overall perceptions of the use of English among participants

Stakeholders, facilitators and students expressed mixed feelings about the use of English as the medium of instruction for the nutritional epidemiology teaching programme. English was described by some facilitators and students as being an “international language” and the “language of science” which was perceived as essential to achieve high-quality research. Most participants mentioned that using English provided KSPH staff and students with more opportunities to access and interact with the scientific community. This included the opportunity to present research findings at international forums and scientific conferences, and authorship of peer-reviewed publications. In particular, one facilitator mentioned that nutrition is not a large field, and most published books and papers are written in English, so that students learning in English were able to access high-quality literature.

So, being obliged to follow classes in English and for some of them (students) to write a thesis in English is kind of a good preparation, especially because we want them to be able to conduct independent research. So, for them you cannot be a researcher, you know, without mastering the English language. (Facilitator 3, KSPH)

Furthermore, many students believed that improved English skills would advance their careers as academics or researchers, and this encouraged them to learn the language. It was also mentioned that increasing numbers of job opportunities included English as a requirement.

It was difficult. Different. A challenge. But good experience because now for all jobs that you are looking for they ask [if] you know English. But it is difficult because we are not an English-speaking country. That’s all our problem. (Student 4, master’s student)

Another key benefit expressed by participants was that using English allowed for the participation of senior researchers from the partner institutions (UiB and UKZN), who were English-speaking and would otherwise have been unable to participate. As a result, staff and students from KSPH could benefit from teaching and mentoring from international partners who were perceived to have high-quality skills and competencies.

… An additional advantage was that the course had an English component to it; the courses were facilitated in English due to the fact that there were professors, especially during the first 3 years of the project, who came from Norway and South Africa. They played a great role through their experience in providing guidance and support through the process of facilitation and supervision. These external facilitators were paired with the local facilitators and that contributed in sharing experiences and in the process, strengthening the school capacity. That was not only a plus for us but also for our students, because it provided an extra motivation to the students to know that they will enrich their English skills in the programme. (Stakeholder 4, KSPH)

However, many participants raised concerns about the use of English, stating that it created a barrier to participation for many stakeholders, facilitators and students, such that only those with pre-existing high-level English skills were able to benefit from the GROWNUT programme. Use of English created communication barriers between many of the role players, leading to a lack of inclusivity and ownership of the programme at KSPH. Facilitators and managers from Kinshasa who were not confident English speakers were unable to contribute their skills to the programme or engage with international partners, thus limiting joint planning and decision-making across the school, which sometimes undermined the success of the partnership.

Some (facilitators and stakeholders) of them were like just not, like at ease with speaking or interacting in English. You know when you interact, for emails at least you can copy, paste and you have Google translators, etc., but talking like this as we do is still an issue here in the School [of Public Health], Kinshasa for some of the supervisors. (Facilitator 1, KSPH)

### Entry requirements for students

Applicants for the new Master in Nutritional Epidemiology programme were informed that English would be the medium for teaching and learning. Applicants undertook an entry examination which included an English assessment; however, applicants who performed well overall could be accepted despite performing poorly in the English assessment. Students reported that they were surprised at the use of English because French is the medium of instruction in most educational institutions in DRC. However, they reported that this did not affect their enthusiasm to enrol in the programme, and some students prepared themselves by taking an additional English course ahead of the language examination.

I had to take English classes before attending the programme because I was informed that the course would be taught in English, so I had to prepare myself in advance (Student 10, master’s student)

Before beginning their studies, students undertook a 2-week (60-h) course in scientific English and a further 30-h English course for nutritionists, provided by KSPH to improve their English skills. Most students reported that the duration of the English course was too short for students whose English was poor, and did not adequately equip them with required skills to understand and participate during classroom teaching.

We have two courses here in English, I think this is a joke, you cannot learn English in 2 weeks and become fluent, no. What is the aim of this, sometime we are talking … you know your English course, just spelling banana or potato, we are joking [about it] but that is the message, we have to keep English but we have to change the strategies. (Student 12, PhD student)

### Teaching and learning in English

In the teaching and learning environment, the requirement to use English as the medium language was very challenging for both students and facilitators. Students reported that they struggled to understand what was being taught in class, including both the language and the concepts. This was particularly challenging when being taught by international facilitators, who often spoke very fast, used technical terms, sometimes with an unfamiliar accent, and did not fully consider that students were not used to the language.

The negatives of the method are that sometimes English becomes a challenge to understand and if one does not know how to ask for an explanation, the professor will assume that everything is clear and will continue. Sometimes you are present but miss some points. (Student 6, master’s student)

In addition, the language barrier limited the interaction between students and facilitators and reduced student participation during classroom teaching. Many students lost confidence expressing themselves in English early in the programme, with some reporting that they spent the whole day in class without understanding anything of what was taught.

Sometimes you are asked a question in English but you do not respond because you did not understand what the question is about and sometimes you have answers but you do not know how to speak. (Student 10, master’s student)

Some facilitators expressed concern that the language barrier prevented students from gaining the required knowledge. The requirement to understand academic content at the master’s level while also learning in a new language placed a double burden on students.

With poor language knowledge and it has contributed to low performance of the students because they are trained in a language that they are not mastering. (Facilitator 9, KSPH)

Concerns around students’ performance were raised by facilitators, who stated that poor English language skills may have caused students to underperform academically. Facilitators felt that some students did not fully understand the language; as a result, some failed the subject or produced poor-quality work. One facilitator stated that it was difficult to determine the reason for the poor results.

In Kinshasa, they faced a lot of challenges, those students, in particular they were asked to write in English and to learn in English, which was like their fourth language. Many students struggled with that, making it quite difficult to tell whether the poor quality of some students’ work was related to poor understanding, maybe poor teaching, or whether it was simply that the language barrier was too great and people were not able to get over that. I think we did a very good job at identifying challenges, making plans to try and overcome those challenges. (Facilitator7, UKZN)

In order to address concerns about students’ understanding of English, those facilitators who were able to speak both languages used both English and French when teaching, which made it easier for students and facilitators to engage about the subject. One facilitator stated that when he was teaching a difficult module, English added more pressure on students as they had to master both the language and the subject, so the facilitator used both English and French when teaching, and allowed students to speak in French.

I am teaching [subject]. As you know, to start with, this is a subject most people do not like because it is difficult. So it is, per se, even if it is taught in the language that you master, it is difficult. So, adding to that a different language, you know, it just makes it more difficult. So, what I was doing was ok, I would start speaking in English, ask if they have understood. If they do not, ok, I would not hesitate to translate into French, make sure that they really grasp the concept of what we are about to do. (Facilitator 3, KSPH)

English was a challenge for KSPH-based facilitators who were responsible for teaching some modules but were unable to speak English and therefore conducted their lecturers in French.

Personally, I was not teaching in English, as I said I am not fluent in English. However, one could have PowerPoint slides in English, as some concepts cannot be easily translated into French. So, the only option then is to use the slides in English but speak in French. (Facilitator 4, KSPH).

Teaching in French was considered a backwards step by some facilitators who taught in English. As one facilitator stated, facilitators teaching in French adversely affected the progress of students learning English. For the first 2 years of the programme, international facilitators travelled to the DRC for collaborative teaching with local facilitators to assist with conducting classes in English. However, this stopped when political unrest prevented travel, thus reducing the modules that could be taught in English.

The challenge is to keep the use of English all the way through the programme, because as I was saying, some colleagues were reluctant to use English, although in the selection of the teachers we were selecting the teachers because of their practise of English. (Facilitator 8, KSPH)

### Supervision and thesis writing

Most students had two supervisors, a main supervisor from KSPH and a co-supervisor from an international partner institution. Use of English during the research component and for thesis writing was viewed both positively and negatively by students and supervisors. Students felt that having both an English- and French-speaking supervisor was beneficial and allowed them to practice both spoken and written English. Local supervisors provided a bridge and supported students with the challenges of communicating with international supervisors.

It helped me, it helped me too much. One supervisor was a French speaker, the other one an English one, we had to write our thesis in English, you see. So, I was like in the middle and having two information’s, English and French, so it was helpful. (Student 11, master’s student)

Communicating by email with international supervisors was a challenge, and most students would have preferred face-to-face supervision. However, email communication did provide a further opportunity to practice reading and responding to supervisors’ comments in English.

There were some students who experienced face-to-face interaction with the international supervisor and gained more exposure to the language. This was particularly mentioned by PhD students who had the opportunity to spend time in English-speaking countries and were able to improve their English skills through their interaction with the co-supervisor and spending time in an environment where English was a dominant language.

Secondly, the English language, before I could not speak English fluently. I gained more experience. I have been in South Africa twice … During this time, I improved my English skills. To me English language is important and if today I can speak, it is because I went to South Africa. (Student 9, PhD)

Although writing their thesis in English was difficult, being able to publish manuscripts from the thesis opened learning and career opportunities for PhD students.

Before this program I used to do everything in French as you can imagine, from my elementary school to university, in the DRC we use French as the official language. When I enrolled in this course, we had to do it in English now, publishing, everything is in English. I had to publish first four papers in English for the peer review, I was obliged myself to increase my English skills, my writing, speaking … I am using this skill to do other things with some others at university. (Student 12, PhD).

A concern expressed by students and supervisors was that writing in English increased the time taken for students to complete their thesis, delaying graduation for some students. Further, the language compromised the quality of students’ work as they failed to express their ideas clearly and coherently. At times, supervisors reported being unable to determine whether the poor quality was related to poor language skills or poor quality of the work. Poor writing skills also added pressure to supervisors’ work.

I was spending hours and hours on the language itself, you know, the things that they write, you have to fix them … So, it blocks me so I cannot read, and I have to make sure that that is corrected. So, for those who are using English for the first time to write a dissertation, you know, that level of work, so that is a big challenge. Even when we have gone through all that, it is always necessary to have like editing resources. (Facilitator 3, KSPH)

Writing a thesis in English proved difficult for some students. Of the total 40 students who completed the programme, six were unable to write their dissertation in English and wrote in French.

I can’t say that I have got all words in English or all information in grammatical ways to write a sentence in English, it was not that easy for me to get all those sentences in English. Yes, this was a negative point because we are not English speakers, we have to write it, a thesis is a book, we have to write it in a language that we are not really comfortable in. (Student 11, master’s student)

A common view among facilitators and supervisors was that the aim of encouraging students to learn and understand English was so that they could develop skills to enable them to publish their work. Although students were able to improve their writing skills with the support of their supervisors and write their dissertations in English, the quality of English writing was inadequate to write for publication. Further support for writing skills would be needed for research to be publishable.

We want them to be able to publish an article, at least from the dissertation itself. So, getting to that level, you know, for them is really difficult but, you know, they have to do it. They have to do it because they are being trained not only to be nutritionists or [inaudible] but also to be researchers, to write, to communicate in writing. (Facilitator 3, KSPH)

### Participants’ recommendations

Students and facilitators requested that the English course be extended beyond 2 weeks to include language teaching throughout the programme, and to increase exposure of students to an English-speaking environment. It was suggested that more visits from international facilitators as well as regular visits to English-speaking countries would increase exposure to the language and improve their English language skills. In addition, participants suggested more workshops for students and academic staff together with international institutions for skills development in scientific writing.

A common view among participants was that the programme could have made greater use of bilingualism, using both English and French to minimize challenges of the language barrier. This was frequently emphasized by students, facilitators and supervisors who reported that the bilingual system worked for them, and it would be of benefit for the GROWNUT programme to implement it.

The issue of language, maybe I am, this is my opinion, I am of the opinion that they can use both languages, that a professor can teach English and French. So, it is good rather than saying only English or only French. (Facilitator 3, KSPH)

## Discussion

Our findings showed that most participants were strongly in favour of English language use in principle, believing that this would open doors and provide opportunities for individual students, for academic staff, for the school of public health and for the country as a whole. EMI provided the opportunity to participate in an international collaboration, and this was appreciated by staff and students at KSPH with wide-ranging benefits including access to the scientific community, improved credibility of the research and production of a cadre of highly skilled researchers. However, this is contrasted with the reality of participants’ experiences, where some students described being left behind and being unable to achieve the knowledge required because of inability to understand the teaching, leading to poor-quality work, so that some students were unable to write their thesis in English. Supervisors became frustrated because they were unable to distinguish poor quality of work from poor quality of English. In addition, there was limited participation in the programme at KSPH, and non-English speaking staff members at KSPH were excluded, so their skills could not benefit the programme. A single EMI programme may not be sustainable in an overwhelmingly French-speaking institution, unless there is strong demand and support from staff and students.

Our findings mirror what is reported in the international literature about the complexity of choosing a language of instruction and are similar to opposing views being expressed [14, 15, 19, 28, 33, 34]. Low English proficiency among students leading to poor academic performance is the main challenge in every EMI programme [14, 15, 33]. Students face the double challenge of having to develop academic English proficiency and content knowledge in diverse subjects which can undermine the overarching aim of producing a high-quality cadre of graduates and academics who will compete globally. For an EMI programme to be successful, it is essential to ensure that students’ and lecturers’ level of English proficiency is adequate to promote excellence in learning the subject content [21, 31].

GROWNUT included an English assessment and 2-week English course, but it is very clear from our findings that this was inadequate, and poor English proficiency among students and some facilitators remained a barrier to success. Going forward, the English assessment needs to be reviewed to ensure that it provides a valid measure of how effectively potential students can communicate and write in English. In addition, support for English speaking and writing needs to be strengthened throughout the programme. In order to make the programme more inclusive, we could consider providing students with online self-study resources to improve their English ahead of enrolment assessments, to avoid excluding promising candidates solely on the basis of poor English performance. Online English courses could also have provided ongoing support for English language development throughout the programme at minimal cost. English language editing was provided to some students in the later cohorts, but could have further supported students to achieve high-quality written work throughout the programme and, in particular, for their thesis. In order to promote equity in an EMI programme, it is essential that strong support for English language skills is built into the programme for all potential participants and lecturers [22].

Inadequate English proficiency of some lecturers was another barrier mentioned by participants, particularly because participation of international facilitators was limited. English skills for local facilitators was not assessed in this programme, and this was a shortfall. Research suggests that teaching in a new language is very demanding, and supporting English skills of lecturers and facilitators is just as important as supporting the students [29]. Several studies have identified that a major hindrance of EMI progress is the lack of English proficiency among lecturers [19, 29, 35]. When lecturers are proficient in English they are able to communicate effectively, which enables learning and understanding of concepts and content knowledge, and stimulates students’ intellectual growth [36]. Defining the level of English language skills required by lecturers and ensuring that this is achieved before teaching is started could also be a tool to improve the quality of English language teaching. Support and resources for learning English should not only be aimed at students but also should be provided to any staff at KSPH who were interested in participating. Establishing high levels of competency in English among teachers at HEIs in the short term can strengthen a bilingual teaching approach and improve sustainability in the longer term.

Language is a two-way street where both teachers and students need to possess necessary language skills to be able to participate effectively in EMI classes. When international facilitators were teaching in English, this had its own challenges, where students described becoming overwhelmed, being unable to follow the lectures and lacking confidence to express their views when being taught by English-speaking lecturers. This undermined the purpose of interactive teaching and learning. As Nyika (2014) mentioned, effective interaction between teachers and students is critical because it allows students to express their enquiries, and teachers can explain with appropriate examples or demonstration [21]. Although some students felt intimidated by English-speaking lecturers, particularly at the beginning of the EMI programme, these facilitators also encouraged students to increase their English skills and provided students with a platform to practice the language on a daily basis.

Although EMI is an approach to making English language academic resources more accessible to students, the challenges expressed highlight the importance of considering other approaches, particularly in the age of globalization, where technologies supporting translation are available. For media where academics exchange knowledge, for example, academic journals and conferences, resources and technology-enhanced approaches should be provided to make these more accessible to a wider non-English-speaking audience. These technologies could also provide resources for students learning in English to support bilingualism.

Both academic staff and students expressed that being given the opportunity to spend time in an English-speaking environment had a very beneficial effect on their English skills. A possible solution to improving English skills would be for students and academic staff to be given opportunities to practice language skills with competent English speakers inside and outside of the classroom [34]. This could be achieved by travelling to English-speaking countries, attending conferences, or short-term placements in partner institutions or using online resources. Belhiah and Elhami (2014) suggest the use of communicative language teaching (CLT) for students, where students get the opportunity to interact frequently in the language that they seek to learn, based on the view that communication and interaction is crucial for language acquisition [31]. The authors suggest that communicative situations such as interacting with teachers, other students, administrators, advisors and other reliable contexts would provide students with ample opportunity to practice English. Although such opportunities were provided to GROWNUT students, more emphasis could have been placed on English language communication for both students and facilitators.

Applying a bilingual system is another approach that has been suggested by a number of academics, policy-makers and linguists as a way of improving the EMI programme [16, 28, 30, 33, 37], and was also suggested by participants. In some courses, this facilitated learning by making it easy for students to engage with facilitators and to understand the content being taught. Macaro (2014) acknowledges the relevance of code-switching (CS) during learning in a classroom, where lecturers switched to the home language when explaining technical terms, checking students’ comprehension and correcting grammatical errors, particularly with students who have limited skills in English [15]. Hiring teachers or facilitators who are competent in both the home language and English language would offer efficient pedagogical and educational usage when switching to the home language during teaching and learning [33].

Academics, linguistics and policy-makers argue that English can only be learnt through being immersed in the language and through continuous listening and speaking in English, while others maintain use of the home language is advantageous when students have limited proficiency in English [19]. However, the main question is whether teaching should be done exclusively in English, and risk students not understanding the content of the subject, should be done in both languages and risk students not improving their English skills, which would prevent them from competing globally. This is a difficult question to answer, but based on the outcomes of this study and despite the challenges, we believe that the EMI within the GROWNUT project enabled most students to learn both language skills and the nutrition epidemiology content simultaneously. We further believe that GROWNUT has developed a new cadre of expertise in DRC who could be future teachers of the new programme and thus harness their bilingualism to achieve the undoubted benefits of the programme.

Implementation of EMI in countries where English is not the dominant language or common language of instruction creates challenges for teachers and students, especially in the early phases of a new programme. However, in the long term the development of new cadres of scholars with both subject and bilingual language skills makes it possible to overcome many challenges of EMI for future offering of newly developed programmes. This has been the case with GROWNUT where some students have now taken up teaching positions at KSPH. This development allows for greater harnessing of bilingualism, thus allowing future cohorts of students to more easily access the international scientific community and scholarship which remains bound in English. Job opportunities and international collaborations are likewise facilitated. Thorough support must be provided to students and teachers to increase their English proficiency in the early phases, until a critical mass of teachers with high levels of English proficiency are developed. These teachers will also provide support for a bilingual teaching and strengthen sustainability of the programme.

### Strengths and weaknesses of the study

The evaluation methodology represented everyone who was part of the GROWNUT project, giving our study credibility. Using experienced qualitative researchers who were not part of the project allowed participants to express their views freely. Two de-registered PhD students were excluded, although one was interviewed as a master's graduate, but the other deregistered PhD student could have been provided valuable insights into EMI.

A key limitation of the study was that it included only GROWNUT participants; those who were excluded from the programme due to poor English skills did not contribute. Different languages between the interviewer and interviewee was a barrier for some interviews, including use of interpreters and translation of interviews, which may have led to inaccurate interpretation of the data. Bias might have occurred because programme stakeholders and facilitators might have wanted to show the programme in a positive light. Although the interviewers had not participated directly in GROWNUT, they were associated with the project team, and this may have made participants, particularly students, reluctant to criticize the programme. Since this was an internal evaluation, some of the authors in this paper were participants. To avoid conflict of interest, only coded data was shared with other team members. Any identifying information was removed from the data to ensure that participants could not be recognized. Participants had unique study numbers, allowing for anonymity to be preserved.

## Conclusion

The use of international partnerships to develop research skills is strongly aligned with the SDGs, but language barriers are common in such partnerships [10–12] and must be urgently and proactively addressed. Through the use of EMI, this project produced qualified researchers, strengthened the research skills in the DRC and at the KSPH, generated important research and provided long-term benefits for some individual participants. Use of English made it possible for partnering institutions to be fully involved. However, these benefits must be seen in the context of the very real pitfalls highlighted by our study where many student and staff were sidelined, unable to participate fully, and failed to achieve their academic potential. Using EMI is an increasing trend globally [20], and further research is required to understand how to implement this approach so that students and academic staff can achieve the maximum benefits while maintaining the cultural identity associated with the home language.

## Data Availability

All data, transcripts and study tools to support the findings of this study are available from the Centre for Rural Health, UKZN, and will be made available upon reasonable request from the principal investigator or corresponding author.

## Abbreviations

DRC: Democratic Republic of the Congo
GROWNUT: Growing Partnership for Higher Education and Research in Nutritional Epidemiology
NORHED: Norwegian Programme for Capacity Development in Higher Education and Research for Development
Norad: Norwegian Agency for Development Cooperation
UNIKIN: University of Kinshasa
KSPH: Kinshasa School of Public Health
UKZN: University of KwaZulu-Natal
UiB: University of Bergen
CIH: Centre for International Health
CRH: Centre for Rural Health
SONPH: School of Nursing and Public Health
EMI: English as a medium of instruction
HEIs: Higher education institutions
MPH: Master in public health
FELTP: Field Epidemiology and Laboratory Training Programme
HSSREC: Humanities and Social Sciences Research Ethics Committee
NSD: Norwegian Centre for Research Data
CLT: Communicative language teaching
CS: Code-switching
LMIC: Low- and middle-income countries
